# Macrophage-targeted nanomedicine for the diagnosis and management of atherosclerosis

**DOI:** 10.3389/fphar.2022.1000316

**Published:** 2022-09-09

**Authors:** Ping Ping Hu, Shuang Xue Luo, Xiao Qing Fan, Di Li, Xiao Yong Tong

**Affiliations:** ^1^ Chongqing Engineering Research Center for Pharmacodynamics Evaluation, College of Pharmacy, Chongqing Medical University, Chongqing, China; ^2^ School of Pharmaceutical Sciences, Chongqing University, Chongqing, China; ^3^ Department of Thoracic Surgery, Daping Hospital, Army Medical University, Chongqing, China; ^4^ Department of Pharmacy, The Second Affiliated Hospital of Chongqing Medical University, Chongqing, China

**Keywords:** atherosclerosis, macrophage, nanomedicine, diagnostic imaging, therapeutic

## Abstract

Atherosclerosis is the primary cause of cardiovascular diseases, such as myocardial infarction and stroke, which account for the highest death toll worldwide. Macrophage is the major contributor to atherosclerosis progression, and therefore, macrophage-associated pathological process is considered an extremely important target for the diagnosis and treatment of atherosclerosis. However, the existing clinical strategies still have many bottlenecks and challenges in atherosclerosis’s early detection and management. Nanomedicine, using various nanoparticles/nanocarriers for medical purposes, can effectively load therapeutic agents, significantly improve their stability and accurately deliver them to the atherosclerotic plaques. In this review, we summarized the latest progress of the macrophage-targeted nanomedicine in the diagnosis and treatment of atherosclerosis, and their potential applications and clinical benefits are also discussed.

## Introduction

Atherosclerosis is the principal pathological basis of cardiovascular diseases (CVDs), which is responsible for the dominant cause of morbidity and mortality in the world (Virani et al., 2021). Atherosclerosis is a multifactorial, chronic inflammatory disease (Ross, 1999; Tabas et al., 2007), characterized by dysregulated lipid metabolism and plaque build-up inside the arterial wall (Negre-Salvayre et al., 2020). The genesis of atherosclerotic plaques is triggered by focal areas inflammation of the arterial tree, which is induced by the subendothelial retention of lipoproteins and immune cells. Endothelial cells (ECs) are damaged, the permeability of which increases, and a large amount of cytokines and chemokines are released, facilitating monocyte attachment and infiltration. Monocytes differentiate and mature into macrophages, and secrete inflammatory factors. Smooth muscle cells (SMCs) in vascular tunica media differentiate to synthetic phenotype and migrate to intima (Que et al., 2020). With the high level of lipidic contents, macrophages and transformed SMCs ingest and accumulate large amounts of modified low-density lipoprotein (LDL), leading to the formation of foam cells and early atherosclerotic plaques (Katakami, 2018). The disintegration of lipid overloaded foam cells leads to the formation of necrosis. When plaques progress, the vessel wall continues to remodel, resulting in arteries thickening and lumen narrowing. Additionally, migrated SMCs proliferate and actively produce extracellular matrix, contributing to the formation of fibrous cap. Once the fibrous cap is broken, causing rupture of vulnerable plaques and acute thrombotic events (Wilson, 2010). There is no specific drug for the treatment of atherosclerosis in clinics. Conventional treatment is mainly based on lifestyle changes in diet and exercise, several drugs are also routinely used, including inhibiting endothelial dysfunction, lowering lipid retention, reducing inflammation and stabilizing plaques. Statins and notable inhibitors of proprotein convertase subtilisin/kexin type 9 (PCSK9) demonstrate great potential in the clearance of cholesterol-containing LDL particles, reducing the incidence of CVD (Forster et al., 2002; Lieb et al., 2018; Dhindsa et al., 2020). However, these medications can only prevent rapid deterioration, and always have potential adverse side effects. For example, statins, the first-line therapy for atherosclerosis, are reported to have liver and muscle toxicity (Maron et al., 2000), and induce depression, headaches, and some other issues with the gastrointestinal system, skin as well as eyes (Kiortsis et al., 2007; Bełtowski et al., 2009). There are also some safety concerns with the employment of cholesterol-lowering drugs in childhood (Chen et al., 2022). Moreover, inflammation is the primary cause of atherogenesis and plaque destabilization, and much effort has been devoted to the development of novel anti-inflammatory drugs, however, the low bioavailability, poor targeting, and high toxicity of which limited their usage clinically.

During the past few decades, the application of nanotechnology for atherosclerosis management has grown exponentially. Nanoparticles/nanocarriers, with dimensions ranging from 1 nm to 100 nm have become an important tool, which demonstrates particular advantages in the improvement of atherosclerosis diagnosis and treatment ( Qiao H. et al., 2017; Xiao et al., 2017). By attaching the antibodies, peptides, or aptamers to the surface, nanomaterials are capable of specifically targeting the receptors or structures characterized in atherosclerosis (Shi et al., 2011). The extremely small size allows the nanoparticle products efficiently enter into living systems, including animals and the human body. Extensive studies reveal that nanomaterials possess strong encapsulation performance, which can protect the loaded therapeutic agents, such as small-molecule drugs, antibodies, peptides, and even small interfering RNA (siRNA) as well as microRNA (miRNA), from metabolic deactivation until they are delivered to target sites (Wang et al., 2016; Chen et al., 2022), reduce systemic adverse effects (Rosenblum et al., 2018) and improve pharmacokinetic and stability of the loaded diagnostic agents or therapeutic compounds (Lobatto et al., 2011), compared to systemic administration of the drugs alone. Notably, targeted nanoparticles are unusually designed to constitute imaging agents and improve the delivery of therapeutics to the local inflammatory macrophages of plaques, which is the critical cell type involved in atheroprogression.

In this review, the recent advances of various nanomedicine for molecular imaging, clinical diagnosis and intervention of atherosclerosis were summarized, and the application of macrophages in atherosclerotic plaques targeted nanoparticles were specially highlighted. Additional insights regarding the opportunities and challenges of macrophage-targeted nanotherapy in clinical translation are also discussed herein.

## Macrophage biology in atherosclerosis progression

Atherogenesis involves the synergistic interaction between lipid metabolic factors and vascular cell components. Macrophages are one of the most important cells for atherogenesis, and they are critical during every stage of atherosclerosis progression. In the early step of atherosclerosis, activated ECs release chemokines (eg. monocyte chemotactic protein 1, MCP-1) and adhesion molecules (eg., intercellular adhesion molecule 1, ICAM1 and vascular cell adhesion molecule 1, VCAM1), inducing the attachment of monocytes to the arterial vessel wall (Potteaux et al., 2011; Moore et al., 2015). The recruited monocytes then differentiate into macrophages in the local damaged intima and acquire distinct phenotypes under different physiological and pathological stimuli (Hoeksema et al., 2012). M1 is the classical inflammatory macrophage, with the high expression of pro-inflammatory cytokines, and reactive oxygen/nitrogen species (ROS/RNS), while M2 is identified as the alternatively activated macrophage, characterized by the capacity of inhibiting inflammatory responses and promoting tissue remodeling and repairing ( Hoeksema et al., 2012; Jinnouchi et al., 2020 ). Lesional macrophages in human atherosclerotic plaques are constituted of various macrophages with different phenotypes and functions, and the phenotype represents dynamic changes in the plaque tissue (Depuydt et al., 2020). A deep understanding of the mechanism that drives the pro-inflammatory and pro-resolving transformation of lesional macrophages may likely provide new therapeutic opportunities for atherosclerosis.

Extensive studies indicated that M1 macrophages are enriched in progressing atherosclerotic plaques, and avidly ingest modified lipoproteins *via* activated cholesterol-trafficking pathways, such as scavenger receptor A (SRA), LOX1 and CD36, resulting in the generation of “foamy” macrophages in the plaques (Moore et al., 2015). Alternatively, excessive lipids are removed by macrophage transporters such as ATP-binding cassette transporter A family member 1 (ABCA1), ABCG1 and liver X receptor-α (LXRα) ( Moore et al., 2015; Tall and Yvan-Charvet, 2015 ). LXRs have been identified as the main targets for intervention in atherosclerotic cardiovascular disease. By promoting cholesterol efflux in macrophages, atherosclerosis is significantly ameliorated in animal models (Tangirala et al., 2002; Naik et al., 2006; Park, 2014; Westerterp et al., 2016). However, the systemic pharmacological intervention of the traditional drugs does not only affect the target cells and organs but also may induce various adverse reactions. For example, synthetic LXR-targeted therapy is complicated, due to its dual effect of alleviating atherosclerosis and promoting hepatic steatosis and hypertriglyceridemia, which restricts the clinical translation of LXR agonists to human disease (Fessler, 2018). To solve this problem, macrophages targeted therapy arouses great attention, in which cholesterol is transferred directly to adjacent cells *in vitro*, instead of cholesterol efflux depending on ABC transporters (He et al., 2020).

Due to the critical role of macrophages in the progression and regression of atherosclerotic plaques, these cells are one of the most utilized therapeutic targets in managing atherosclerosis. In addition, the non-immune cell-targeted nanomaterials may be primarily taken up within the plaques (Beldman et al., 2019), and various nanoparticles/nanocarriers *via* macrophage-mediated uptake have emerged as promising candidates for diagnostic imaging and therapeutic applications in atherosclerosis.

## Nanoparticles for diagnosis of atherosclerosis

**FIGURE 1:**
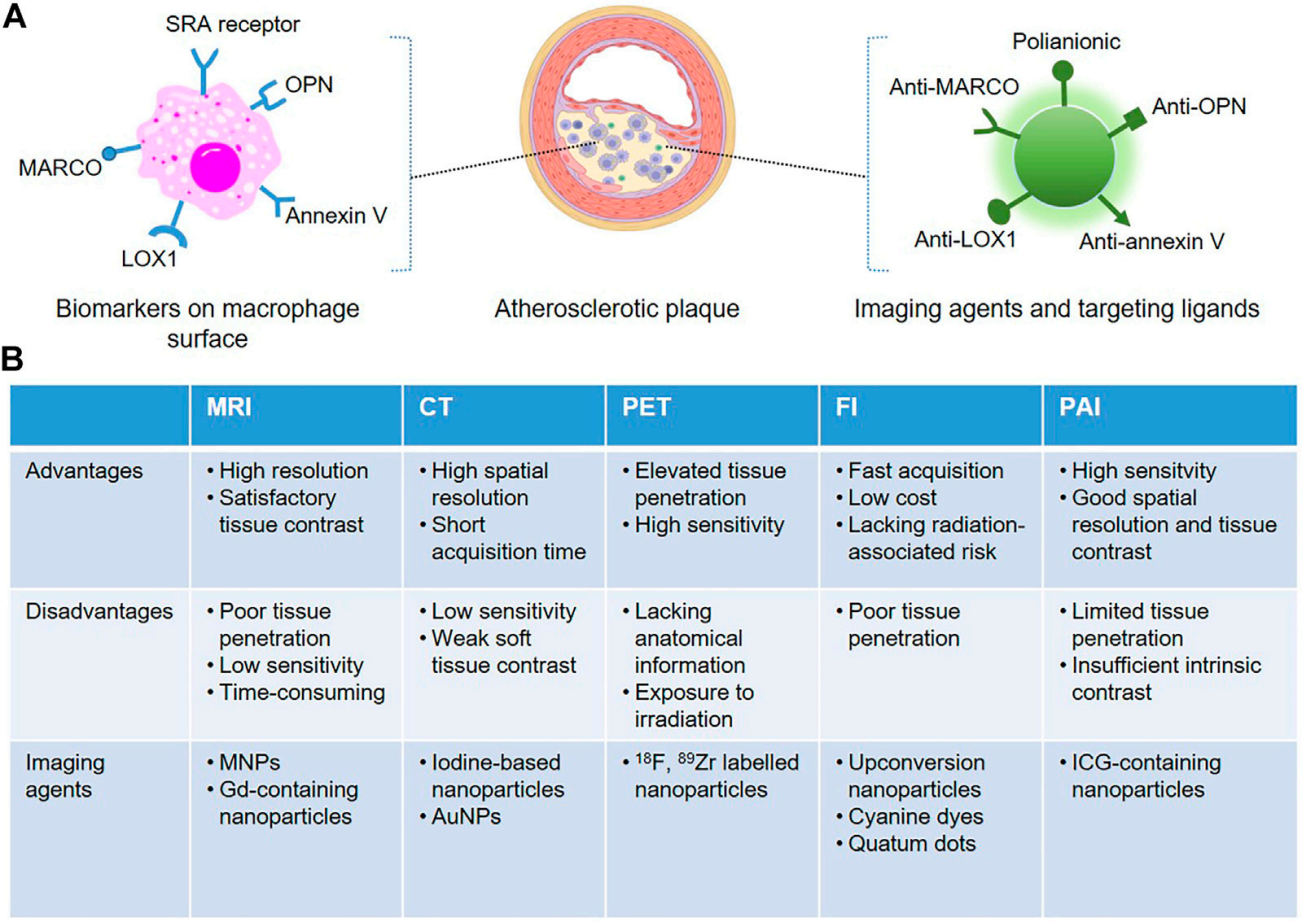
Schematic representation of macrophage-targeted nanoplatforms for non-invasive atherosclerosis diagnostic imaging. **(A)** The commonly used epitopes overexpressed on the surface of lesional macrophages can be specifically recognized by targeted nanoparticle-based imaging agents. **(B)** Advantages, disadvantages and the associated imaging agents of the non-invasive imaging modalities.

An angiogram is the clinical “gold standard” for defining the presence and extent of coronary artery disease, which fails in reliable predicting the sudden rupture of vulnerable plaques. Growing studies reveal that the composition of plaques is the primary determinant of their stability, and unstable plaques are always characterized by an abundance of macrophages and necrotic lipid cores, surrounded by quite thin or disrupted fibrous caps (Finn et al., 2010). “Molecular imaging technology”, a novel image-based technique, achieves to visualize atherosclerotic plaques with high spatiotemporal resolution in real-time in a non-invasive way. With the assistance of robust imaging agents and therapeutic drugs for anti-atherosclerosis, various nanoparticle-based imaging agents, that can specifically target lesional macrophages are designed, and pivotal insight into plaques is obtained. For example, many macrophages-targeted imaging nanomaterials have been commonly applied for the identification of atherosclerosis through magnetic resonance imaging (MRI) (Huang et al., 2012), computed tomography (CT) (Shilo et al., 2012), positron emission tomography (PET) (Phelps, 2000), fluorescence imaging (FI) (Qiao R. et al., 2017), photoacoustic imaging (PAI) (Ge et al., 2020) and combined imaging techniques. Each imaging system displays advantages and disadvantages, and two or more imaging platforms are recently combined to provide more comprehensive information about the pathophysiological process of atherosclerosis. Advances of nanotechnologies have then generated various types of nanomaterials that are robust and reliable for molecular imaging, which are summarized in Figure 1.

MRI, using magnetic iron oxide nanoparticles (MNPs) or Gd-containing nanoparticles as imaging agents, is a common technique for the early diagnosis of atherosclerosis. MRI can provide anatomical images with high resolution and satisfactory tissue contrast, but poor tissue penetration and low sensitivity, since atherosclerosis may occur anywhere (Huang et al., 2012). Macrophages can uptake nanoparticles or other foreign substances *via* pinocytosis, endocytosis and phagocytosis. The latter is the main process for engulfing large particles, while the uptake procedure is distinct depending on the size and nature of the surface coating of nanostructures. For example, dextran-coated superparamagnetic iron oxide nanoparticles (SPIONs) and ultra-small super-paramagnetic iron oxide nanoparticles (USPIONs) are known to be phagocytosed spontaneously by plaque macrophages. However, high doses are always required and the process is time-consuming to achieve a suitable contrast of plaques in the surrounding tissues. Macrophages in atherosclerotic plaque express a high level of SRA, which is involved in atherogenesis and associated with inflammatory responses (Moore et al., 2015), but it is impossible to be found in normal vessels. The substitution of dextran with dextran sulfate leads to magnetic nanoparticles recognized by the SRA receptor, which is reported to be specifically identified by polyanionic macromolecules, inducing an increment of 4-fold in the contrast ratio, in comparison with dextran (Tu et al., 2011). Moreover, some other targeting moieties, such as osteopontin (OPN) (Qiao H. et al., 2017), human ferritin protein cages (Terashima et al., 2011) or annexin V (Cheng et al., 2015) have been widely used to be coated in the surface of SPIONs, that can specifically bound with the epitopes on macrophage surface, facilitating the accumulation of nanoparticles in vulnerable plaques. In clinical studies, MNPs enhanced MRI has been successfully utilized to identify pro-inflammatory macrophages associated with atherosclerotic burdens, especially rupture-prone lesions (Kooi et al., 2003). Of note, magnetic high-density lipoprotein (HDL)-like nanostructures developed by Lüscher et al. demonstrated an atheroprotective effect by promoting reverse cholesterol transport (Lüscher et al., 2014).

CT imaging is one of the most convenient diagnostic imaging modalities in clinical settings. CT as a radiology biomedical imaging tool provides images with superior spatial resolution and short acquisition time to identify macrophages in plaques of coronary arteries, but it is difficult to distinguish different soft tissues (Yu and Watson, 1999; Shilo et al., 2012). The high atomic number iodine (Z = 53) can absorb X-rays effectively and is a present used CT contrast agent clinically. Considering that iodine molecules are easily eliminated by kidney, iodine-based liposomes, polymers and micelles are designed, and the iodine-containing nanomaterials are advantageous to assess pro-inflammatory macrophages of vulnerable plaques in the vascular system (Torchilin et al., 1999; Aviv et al., 2009; Hallouard et al., 2010). However, due to the poor sensitivity of CT, high doses of nanomaterials are required to obtain satisfactory images, which may be toxic and limit their future clinical translation. With recent advances in the development of nanotechnology, gold nanoparticles (AuNPs), with unique chemical stability, biodistribution and biosafety, can be synthesized commercially, and the flexibility in size, shape and surficial functional groups for targeting, makes AuNPs become the next promising generation of contrast agent for CT imaging (Kojima et al., 2010).

PET is an analytical imaging technology, which is particularly advantageous in non-invasive and quantitatively characterizing inflammatory macrophage-mediated atherosclerosis. PET displays excellent tissue penetration and high sensitivity, but anatomical information is not available, which is always coupled with MRI or CT imaging modalities as a consequence. To eliminate the false positive caused by traditional radioactive tracers (eg., ^18^F-fluoro-2-deoxyglucose), which can be avidly taken up by both macrophages and cardiomyocytes (Lobatto et al., 2011), various nanomaterials labeled with PET radioactive tracers were designed. For instance, ^18^F-labeled polyglucose nanoparticles, which can be identified by lesional macrophages with high efficiency, were developed to monitor plaques in animal models (Keliher et al., 2017). Additionally, nanoparticles labeled with ^89^Zr have a long half-life, which facilitates the prolonged monitoring of macrophages (Pérez-Medina et al., 2016).

Fluorescence imaging technique provides a higher spatial resolution compared with other clinical imaging platforms. By detecting fluorophore emission spectra using fluorescence microscopy and derivatives, fluorescence imaging is fast, cheap and some functionalized fluorescent nanomaterials with good physicochemical properties have been tested to assess the status of macrophage-rich plaques in animal models. Emission spectra of currently used nanoparticle-based fluorescent probes are mainly in the near-infrared region to generate deep tissue penetration. Qiao et al. constructed NaGdF_4_:Yb, Er@NaGdF_4_ upconversion nanoparticles, and OPN antibodies were coated on the surface of the nanoparticles, which can specifically target the foamy macrophages in vulnerable plaques (Qiao R. et al., 2017). The resulting probes were then injected intravenously, fluorescence as well as MRI images are acquired to confirm the diagnostic application of the probes in distinguishing rupture-prone plaques from stable plaques *in vivo*. In a recent study, Flores and others pointed out that single-walled carbon nanotubes (SWNTs) can be robustly and preferentially taken up by macrophages, compared with non-phagocytic cells (Flores et al., 2020). SWNTs were then conjugated with a cyanine dye (Cy5.5), and a small-molecule inhibitor of SHP-1 was loaded, which achieved the diagnostic imaging of macrophages in atherosclerotic plaques and accurate delivery of therapeutic agents. In addition, a novel near-infrared (NIR) fluorescence nanoparticle, which can be specifically taken up by macrophages and fluorescence turns on in presence of a macrophage-associated enzyme, was engineered and show great potential in visualization the vulnerability of atherosclerotic plaques (Narita et al., 2019).

PAI Upon combining optical imaging with high sensitivity and ultrasonic imaging that has relatively deep tissue penetration, PAI, as a new non-invasive biomedical diagnostic platform, is applied to assess the plaque composition of arteries with atherosclerosis. Besides providing morphological information with good spatial resolution and tissue contrast, some endogenous contrast such as lipid and exogenous contrast (nanomaterials or organic dyes) are always involved in PAI, helping to distinguish disease tissues from normal ones (Sun et al., 2016; Sun et al., 2017; Zhou et al., 2019). With the emergence and rapid development of NIR nanoprobes, Ti_3_C_2_ nanosheets/indocyanine green (Ti_3_C_2_/ICG) nanocomposites were designed as PA nanoprobes, and similar to other targeted nanoprobes, the anti-OPN antibody was conjugated on the surface, and the resulting Ti_3_C_2_/ICG nanoprobes specifically accumulate in foam cells and vulnerable plaque tissues in mice, endowing the dual-modality imaging strategy for the precise diagnosis of atherosclerotic plaques (Ge et al., 2020). In another study, bovine serum albumin (BSA) based self-assemblies (BSA-Cy-Mito) displayed strong photoacoustic responses to GSH/H_2_O_2_ simultaneously (Gao et al., 2019). Systemic administration of the redox-responsive nanoprobe was further achieved to early identify the rupture-prone plaques in mice.

## Nanoparticles for therapeutics of atherosclerosis

As mentioned above, intimal proinflammatory macrophages are the primary contributors in all stages of the atherogenic process, which are participated in atherogenic inflammations and aberrant lipid metabolism and subsequent generation of foam cells. As a result, many exogenous small molecule drugs and biomolecules (eg., siRNA, miRNA, circRNA, peptides and proteins) have great potential to improve the functions of plaque macrophages through activating or silencing the key signaling pathways in macrophages. Traditional drugs always have defects of short half-lives, side effects and inefficiency of oral administration, which greatly limited their applications. Interestingly, the permeability of vascular endothelium is found to increase in evolving atherosclerotic plaques, facilitating more lipoproteins or some other small particles such as nanoparticles/nanocarriers to enter into the intimal layer and atherosclerotic lesions (Atukorale et al., 2017). Since the first therapeutic application of nanoparticles in a clinical study (Cho and Han, 2018), nanotherapeutics in atherosclerosis have fueled interest to improve drug stability, precise targeting, and efficacy (Flores et al., 2019). In this section, we outline and discuss the latest developments of macrophage-targeted nanomaterials for atherosclerosis treatment, and the potential clinical prospect of these treatments is also evaluated. The primary macrophage-associated pathological processes of atherosclerosis such as macrophage accumulation and proliferation, obligatory atherogenic inflammations and excessive lipid accumulation are considered the compelling targets of anti-atherogenic nanotherapeutic agents.

Suppression of accumulation and proliferation of macrophages Experimental data indicates that impaired vascular endothelial function drives monocyte adhesion and infiltration to the arterial wall, resulting in macrophage accumulation in plaques (Jan et al., 2020). Besides, survival/proliferation of lesional macrophages may affect their overall content, and macrophage apoptosis amplifies inflammations and triggers further monocyte adhesion and recruitment (Gautier et al., 2009; Cheng et al., 2015). As a result, inhibition of accumulation and proliferation of macrophages becomes a promising therapeutic target, which can not only decrease the overall content of plaque macrophages but also prevent the rupture of plaque and acute atherothrombotic events (Lutgens et al., 2010; Potteaux et al., 2011). In a preclinical study, Lameijer et al. incorporated a small molecule inhibitor with reconstituted HDL, which blocks the interaction of CD40 and tumor necrosis factor receptor-associated factor 6 (TRAF6) (Lameijer et al., 2018). A 1-week treatment with the proposed nanocomposites (TRAF6i-HDL) demonstrated significant anti-plaque inflammatory effects, by decreasing monocyte recruitment as well as the number of lesional macrophages in atheroprone *ApoE* ^*−/−*^ mice. Of note, the safety of HDL-based nanoimmunotherapy has been established in both mouse models and non-human primates, suggesting their promising therapeutic applications for clinical transformation. Considering the important recruitment effect of vascular ECs to monocytes, Dr. Nahrendorf’s group (Sager et al., 2016) developed siRNA encapsulated polymeric endothelial-avid nanoparticles to simultaneously silence five major endothelial adhesion molecules, and the five-gene combination RNAi reduced the migration of macrophages into plaques in *ApoE* ^*−/−*^ mice after myocardial infarction. In another study, lipid polymer nanoparticles encapsulated with siRNA oligonucleotides were systemically injected into a mouse model of myocardial infarction by the same group (Krohn-Grimberghe et al., 2020). The nanoparticles mediated the silencing of MCP-1 in bone-marrow-derived ECs thereby inhibiting leukocyte release from the hematopoietic niche, reducing monocyte supply to plaques and attenuating heart failure in mice.

Besides inhibiting monocyte recruitment to reduce the lesional macrophage content, suppression of macrophage proliferation and apoptosis in atherosclerotic plaques using nanomaterials also serves as promising therapeutic avenues to stabilize vulnerable plaques. For example, localized delivery of anti-atherosclerotic drug rapamycin using biomimetic nanocomplexes, cloaked with leukosomes (Boada et al., 2020) or red blood cell membrane (Wang Y. et al., 2019), attenuated the progression of atherosclerosis significantly by suppressing macrophages proliferation within plaques and reducing key proinflammatory cytokines. Moreover, encouraged by the superior therapeutic effect of photosensitizers by producing cytotoxic ROS and inducing extensive macrophages apoptosis in artery atheroma in mice under 650 nm light, various photothermal agents such as SWNTs (Kosuge et al., 2012), silica-AuNPs (Kharlamov and Gabinsky, 2012), Au nanorods (Qin et al., 2015) and MoO_2_ nanoclusters (Wang X. et al., 2019) were designed to reduce atherosclerotic plaque burden in preclinical studies. These nanoparticles with extremely high photothermal conversion efficiency and biocompatibility can be preferentially engulfed by pro-inflammatory macrophages. However, their treatment outcome varies in distinct stages of atherosclerosis, which still need tremendous effort (Tabas, 2005).

Anti-inflammatory therapies Atherosclerosis is widely considered a chronic inflammation-related disease, highlighting inflammation dampening as an essential therapeutic strategy. However, systemic administration with traditional anti-atherosclerotic drugs or anti-inflammatory cytokines always fail to reduce the serum level of pro-inflammatory cytokines, and great efforts have been made toward targeted delivery of the therapeutic agents to atherosclerotic plaques to effectively reduce inflammation and their adverse side effect during the past two decades. For example, precisely targeted delivery of interleukin 10 (IL-10), an anti-inflammatory cytokine, utilizing biodegradable controlled-release polyesters (PLGA) (Kamaly et al., 2016) or cRGD peptide conjugated pluronic based nanocarriers (Kim et al., 2020), is more potent in solving inflammation than free IL-10, which leads to increased fibrous cap thickness and decreased necrosis in an atheroprone mouse model. Wu and collogues molecularly engineered 5-aminolevulinate hydrochloride (HDL) electroporated M2 macrophage-derived exosomes (HAL@M2 Exo) with inherent inflammation tropism for the effective administration of atherosclerosis (Wu et al., 2020). In this study, the superior anti-inflammatory effect of M2 macrophages endows HAL@M2 Exo promoting the secretion and release of anti-inflammatory cytokines to alleviate the deterioration of the disease. Simultaneously, extra anti-inflammatory CO and bilirubin were generated with the employment of HAL for heme biosynthesis, therefore boosting the therapeutic effect in a mouse model. Moreover, some other targeted delivery platforms that can reduce the secretion of inflammatory cytokines of macrophages in plaques are also developed for atherosclerotic intervention. For example, MCC950, an NLRP3-inflammasome inhibitor, was delivered using platelet-derived extracellular vesicles, intravenous administration of which can selectively target inflammatory cells and reduce local inflammation and plaque size (Ma et al., 2021). A similar anti-inflammatory effect is reported by nanoparticles-based macrophage targeted delivery of methotrexate in mice (Stigliano et al., 2017; Francesco et al., 2020). Lastly, given the crucial role of ROS for lipids oxidative modification, activation of vascular cells especially ECs and macrophages during atherosclerosis progression, a series of superoxide dismutase mimics such as Cerium (Ce) (Wang et al., 2022) and cyclodextrin (Guo et al., 2019) based nanoparticles are synthesized, which show desirable efficacies to weaken the plaques *via* synergistic ROS scavenging and foam cell inhibition.

Lipid lowing therapies Lipid metabolism disorders induced lipid excessive deposition in macrophages and foam cell formation is a central pathological feature of atherosclerosis, and promoting reverse cholesterol transport (RCT) from foamy macrophages shows great potential to prevent foam cell formation in atherosclerotic plaques (Tangirala et al., 2002; Naik et al., 2006; Rosenson et al., 2012; Park, 2014). HDL, known as good cholesterol, is a type of endogenous lipidic nano-sized particle, constructed by cholesterol, phospholipids and apoA-I. HDL content is widely considered a biomarker of RCT-based cholesterol efflux from foamy macrophages to the liver. Given the antiatherogenic function of HDL, HDL-like nanoparticles are also of considerable interest in atherosclerosis treatment (Lüscher et al., 2014; Banik et al., 2016; Lameijer et al., 2018; Wu et al., 2020). For instance, a biodegradable HDL nanoparticle-based platform was constructed by Marrache and Dhar (Marrache and Dhar, 2013), which displays excellent biocompatibility, stability and nonimmunogenic properties, and is promising for future clinical translation to detect early plaques and prevent the progression of vulnerable plaques. The same group further optimized the HDL-mimicking polymer-lipid hybrid nanoparticles for cholesterol binding and efflux (Banik et al., 2017). The proposed HDL-mimetic NPs with superior M2 macrophage specificity as well as mitochondria targeted effect, can significantly accumulate in the heart and aorta of mice and deliver excess cholesterol to mitochondria to promote its metabolism.

Besides HDL-mimics-based nanoparticles, the upregulation of ABCA1 or ABCG1 expression is another important potential pathway to prevent excess lipid deposition in atherosclerotic foam cells (Rothblat and Phillips, 2010; Westerterp et al., 2016). Accumulating studies demonstrate the therapeutic effect of miRNA-based oligonucleotides in atherosclerosis regression by targeting ABCA1 and ABCG1, such as miR-33, miR-26, miR-206, et al. (Feinberg and Moore, 2016; Nguyen et al., 2019). Chitosan (Nguyen et al., 2019) and cyclodextrin-based nanoparticles (Li et al., 2020) can efficiently deliver efflux-promoting miRNA mimics to macrophages, regulate cholesterol metabolism and substantially reduce lesional burden in an atheroprone mouse model. Additionally, activation of LXRs is also reported to be capable of promoting the removal of cholesterol by upregulating ABCA1 and ABCG1 (Naik et al., 2006). However, systemic LXR activation, which can promote the transportation of cholesterol to the liver and reduce the inflammatory response, is also known to be liver toxic (Fessler, 2018). Biodegradable collagen IV-targeted polymeric nanoparticles encapsulating LXR agonists GW3965 (Col IV-GW-NPs) are reported to specifically target atherosclerotic plaques in *Ldlr* ^−/−^ mice (Yu et al., 2017). Compared to non-targeting encapsulated GW, 5 weeks of administration of the nanoparticles (Col IV-GW-NPs) to pre-existing plaques, lesional macrophage content was significantly reduced, without altering hepatic lipid metabolism during the treatment period, which makes the novel targeted nanoparticle-based system promising for efficient delivery of therapeutic agents to combat lipid accumulation and atherosclerotic inflammation.

## Conclusion

Over the past decades, much effort has been devoted to provide a comprehensive understanding of atherosclerosis pathophysiological processes, and the rapid development of innovative nanoparticles and nanotechnologies fueled the research of nanomedicine-mediated strategies for preclinical diagnosis and administration of atherosclerosis. In this review, we extensively summarized various nanoscopic therapies to diagnose and combat atherosclerosis, as well as the related ischemic CVDs. Especially, lesional macrophage targeted nanomaterial-based atherosclerotic imaging, diagnosis and therapeutics were highlighted. However, since there is a high degree of overlap between host defense and macrophage-involved mechanisms during atherosclerosis progression, the design of nanomedicines without interfering immunity is quite difficult. On the other hand, current imaging techniques remain to fail to precisely identify the composition of lesions or rupture-prone plaques. Furthermore, most atherosclerosis associated biomarkers mentioned above always overexpress only under particular biological conditions, which is a huge setback to the application of the active targeting approaches. Although obvious challenges remain existing, nanomedicine-based approaches show tremendous potential in preclinical settings of atherosclerosis. We believe they will be eventually employed diagnosing and managing patients with cardiovascular disease in the future.
